# Variation in staff perceptions of patient safety climate across work sites in Norwegian general practitioner practices and out-of-hour clinics

**DOI:** 10.1371/journal.pone.0214914

**Published:** 2019-04-10

**Authors:** Ellen Catharina Tveter Deilkås, Dag Hofoss, Elisabeth Holm Hansen, Gunnar Tschudi Bondevik

**Affiliations:** 1 The Norwegian Directorate of Health, Oslo, Norway; 2 Health Services Research Unit, Akershus University Hospital, Lørenskog, Norway; 3 Lovisenberg Diaconal University College, Oslo, Norway; 4 University of South-Eastern Norway, Notodden, Norway; 5 Research Group for General Practice, Department of Global Public Health and Primary Care, University of Bergen, Bergen, Norway; 6 National Centre for Emergency Primary Health Care, Uni Research Health, Bergen, Norway; Public Library of Science, UNITED KINGDOM

## Abstract

**Introduction:**

Measuring staff perceptions with safety climate surveys is a promising approach to addressing patient safety. Variation in safety climate scores between work sites may predict variability in risk related to tasks, work environment, staff behavior, and patient outcomes. Safety climate measurements may identify considerable variation in staff perceptions across work sites.

**Objective:**

To explore variation in staff perceptions of patient safety climate across work sites in Norwegian General Practitioner (GP) practices and Out-of-hours clinics.

**Methods:**

The Norwegian Safety Attitudes QuestionnaireAmbulatory Version (SAQ A) was used to survey staff perceptions of patient safety climate across a sample of GP practices and Out-of-hours clinics in Norway. We invited 510 primary health care providers to fill out the questionnaire anonymously online in October and November 2012. Work sites were 17 regular GP practices in Sogn & Fjordane County, and seven Out-of-hours clinics, of which six were designated as “Watchtower Clinics”. Intra–class correlation coefficients were calculated to identify what proportion of the variation in the five factor scores (Teamwork climate, Safety climate, Job satisfaction, Perceptions of management, and Working conditions) were at work site-level.

**Results:**

Of the 510 invited health care providers, 266 (52%) answered the questionnaire. Staff perceptions varied considerably at the work site level: intra–class correlation coefficients (ICCs) were 12.3% or higher for all factors except for Job satisfaction–the highest ICC value was for Perceptions of management: 15.5%.

**Conclusion:**

Although most of the score variation was at the individual level, there was considerable response clustering within the GP practices and OOH clinics. This implies that the Norwegian SAQ A is able to identify GP practices and OOH clinics with high and low patient safety climate scores. Patient safety climate scores produced by the Norwegian version of the SAQ A may, thus, guide improvement and learning efforts to work sites according to the level of their scores.

## Introduction

For more than a decade, the landmark report, “An organisation with a memory,” has emphasised how the mindset, values and priorities of employees and management influence patient safety [1]. The report acknowledged that adverse events must be valued as sources of useful information for health care organisations to learn and improve. It concluded that improvement in patient safety depends on how healthcare organisations are able to encourage staff to speak up about hazards, risks and adverse events. This requires that staff feel safe and trust that admitting mistakes and adverse events will not be held against them [2]. Since the report was published, widespread efforts to address safety culture in healthcare organisations have emerged [3, 4]. Most have been related to hospital care, but efforts to address safety culture in primary care have also been noted [5–9].

Safety culture refers to individual and group values, priorities, attitudes, perceptions, and patterns of behaviour that specifically determine an organization's commitment to and management of safety [10, 11]. Typical statements that may reflect staff perceptions of a positive safety culture in healthcare are: “It is easy for personnel in this clinic to ask questions when there is something that they do not understand” or “I am encouraged by my colleagues to report any patient safety concerns I may have”. Lack of acknowledgment and respect between professions are examples of cultural characteristics that may create barriers in the way teams are able to cooperate to reduce risks in patient care [12]. The approach through which leaders facilitate time for teams to define goals, initiate action, reflect and adjust their work processes is another cultural trait that may determine learning processes and team success [2]. Typical questions for reflection are, “What should we learn from this?”, “What can we improve?” and “What should we change?” Reflections may be done on a daily basis, at regular meetings, or related to project milestones [13]. In organisational psychology research, safety culture can be studied by using both qualitative and quantitative methods [14]. A promising approach to addressing variation in safety culture between organisational units is to survey staff perceptions [4]. Valid measurements of staff perceptions are referred to as organisational climates, which are mathematical expressions of how cultural norms in natural social units are enacted, as shown, for example, by leader and members reports on how the organisation generally performs [15]. Valid organisational climate questionnaires are able to identify between unit variation in staff perceptions as well as consensus of staff perceptions within organisational units[16]. Both are measured by intraclass correlation coefficients (ICCs). Organisational climates with diverging perceptions are regarded as weak, with little power to predict staff practices [17]. Measurements of staff perceptions of patient safety culture are referred to as patient safety climates. Variation in safety climate across work sites may predict increased risk related to tasks, work environment, staff behavior, and patient results [16–19]. Considerable safety climate variation between work sites also provides opportunity to direct leadership efforts to where safety climate improvement is most needed in hospitals and primary care. Evidence has indicated that primary care teams’ opportunities for dialogue regarding quality of care is associated with better safety climate scores [7]. The Safety Attitudes Questionnaire–Ambulatory Version (SAQ A) is a validated questionnaire that measures staff perceptions of patient safety climate [18].

To be able to address patient safety climate in GP practices and Out-of-hours (OOH) clinics in Norwegian primary care, we translated and validated the Safety Attitudes Questionnaire–Ambulatory Version (SAQ A) into Norwegian [19]. The study was initiated by the National Centre for Emergency Primary Health Care, which has established seven “Watchtower” OOH clinics to deliver research data [19, 20]. These Watchtower OOH clinics serve 4.6% (226,000 inhabitants) of the nation’s population, in a total of 18 municipalities. They are located in different counties across all four health regions of the country, in Nes, Solør, Arendal, Kvam, Tromsø, Alta and Sotra [20, 21]. They are considered representative of OOH clinics in Norway and were, therefore, included in the sample of work sites in the study. In the first paper, we confirmed and presented psychometric properties for five major patient safety climate factors in Norwegian primary care; Teamwork climate, Safety climate, Job satisfaction, Perceptions of management, and Working conditions (S1 Table). In the second paper we documented significant variation regarding several of these patient safety climate factors across professional boundaries and by gender [22]. In this paper, we will explore to what extent the SAQ A identifies variation in patient safety climate perceptions across work sites in Norwegian GP practices and OOH clinics. The analysis is modelled after a previous hospital study in which we documented that staff perceptions of patient safety climate varied considerably across wards and departments [23].

## Materials and methods

### Sample

The study was conducted both in regular GP practices and OOH clinics in Norway. All 30 regular GP practices in Sogn & Fjordane County were invited to participate in the study. This is one of 19 counties in Norway, with a population of approximately 110,000 in 26 municipalities. The participating GP practices serve a population of 70,000. We also invited all seven Norwegian Watchtower OOH clinics previously mentioned in this paper. To protect the confidentiality of the respondents in our analysis, we only included clinics and practices with at least five health professionals with clinical patient contact. For this reason, we replaced one of the seven Watchtower OOH clinics with the OOH clinic in the neighbouring municipality. The seven OOH clinics participating in our study employed a total of 337 health professionals, of whom 231 were medical doctors and 106 were nurses. They served a total population of 251,000. Seven of the total 30 regular GP practices in Sogn and Fjordane County were not included, as they had less than five employees working clinically. Of the remaining 23 regular GP practices, 17 agreed to participate in the study. These 17 regular GP practices employed a total of 173 health professionals: 85 medical doctors and 88 support medical staff. The professional background of the support medical staff varied and included registered nurses, medical secretaries and bioengineers. In this paper, we use the term “support staff” for this group.

### Data collection

#### Variables, scores and measurements

Two Norwegian versions of the SAQ A were used, one for GP practices and one for OOH clinics, with only minor modifications according to the setting [8]. For instance, in the OOH clinic version, the original SAQ A statement “Medical errors are handled appropriately in this office” was changed to “Medical errors are handled appropriately in this OOH clinic”. Both are 62 item questionnaires where the respondents rate their agreement using a five-point Likert scale. The scores of negatively worded items were reversed, so that higher scores in the data set always indicated a more positive evaluation of the unit’s patient safety climate.

S1 Table presents 29 of 62 items of the SAQ A in the version for GP practices, which corresponds to the measurement model of SAQ that has been tested and validated in a previous study [19]. The formula for the factor score for each individual respondent is (mean value of item scores that belong to the factor—1) *25. As the formula shows, factor scores are calculated by subtracting one from the mean, of all single item scores within a factor, and multiplying by 25, so that the score “1” is transformed to “0”, “2” to”25”, “3” to “50”, “4” to “75” and “5” to”100”. This is to achieve a factor score scale from 0 to 100 [24]. Items not included in the measurement model were intended to facilitate discussions to identify local improvement potential.

#### Survey

In October and November 2012, the SAQ A was distributed electronically to all 510 health care providers in the 24 participating GP practices and OOH clinics. Data were collected using the program QuestBack, whereby the participants responded anonymously. This program automatically sent a reminder to those who had not responded after two weeks. After four weeks, an additional reminder was sent to the administrative key persons in the OOH clinics and regular GP practices, asking them to motivate the clinical staff to participate in the study.

#### Ethical considerations

The study was based on data regarding health care providers’ perceptions of patient safety climate and was approved by the Norwegian Social Science Data Services (Ref.no. 2012/30774)–the governmental agency for protecting survey research respondent privacy according to the Norwegian Personal Data Act [25]. In accordance with the Norwegian Social Science Data Services requirements, all participants received written information about the purpose of the study and were informed that the data would be collected anonymously and treated in confidence.

### Statistical analysis

The study was observational with a cross sectional design where staff perceptions of patient safety climate were surveyed. Responders were nested within two types of workplaces: GP practices and OOH clinics. Our data set was hierarchically structured and we used SPSS to quantify how much staff patient safety scores varied across work sites. The multilevel analysis was based on individual respondents’ results for the five patient safety climate factor scores (S1 Table). Multilevel analysis makes it possible to estimate how much of the variance in the data can be attributed to organisational level variance, which is the work site level of the GP practices and OOH clinics in our study [26]. Organisational level variance was calculated by the intraclass correlation coefficient (ICC): the ratio of the variance at work site level(s) to the total variance in the data. Multiplied by 100 the ICC can be read as the percentage of the total variance in the data set that belongs to the organisational level. An ICC of 10 (%) or more is commonly seen as strong clustering of scores by organisational units. Even ICCs as low as 1 (%) have been declared as indicating design effects that should not be ignored [27, 28]. Two empty models were estimated, one including work-site level and one without this level. The Akaike Information Criterion (AIC), where smaller values means better model fit, was applied to compare the models.

## Results

Of the 510 invited health care providers, 266 (52%) answered the questionnaire: 72% of the support staff (n = 139) and 39% of the medical doctors (n = 124). Three respondents did not provide information on their professional status. The response rate was higher amongst medical doctors in GP practices (55%), than medical doctors in OOH clinics (33%). Corresponding rates for support staff were 73% and 71%, respectively. Basic characteristics of the sample have been previously reported [22]. One work site was excluded from the multilevel analysis, as it returned only one questionnaire.

All five patient safety climate factor scores varied considerably according to work site level. The four patient safety climate factors with most between-work site variability for climate measurements in our study were Teamwork climate, Safety climate, Perceptions of management, and Working conditions. Except for Job satisfaction, all patient safety climate dimensions had ICCs of 12% or higher (Table 1).

**Table 1:** Total variance of patient safety climate factor scores. The scores are partitioned at individual and work site (organisational) levels. The ICC coefficients show percentage of organisational to total variance. The AIC value indicates model fit.

Factor (All factors scaled 0–100)	Teamwork climate	Safety climate	Job satisfaction	Working conditions	Perceptions of management
Total variance	226.66	337.85	234.24	437.85	380.72
Variance at individual level	194.02	282.30	217.68	373.79	334.65
Variance at work site level	32.64	55.55	16.56	64.06	46.07
ICC–percentage of work site (organizational) level variance to total variance	14.4%	16.4%	7.1%	14.6%	12.1%
AIC value of two level model: responders nested within work sites	2061.78	2261.16	2197.48	2349.95	2317.82
AIC value of one level model: not considering work site level random variation	2324.61	2275.47	2199.65	2368.01	2324.61

## Discussion

In addition to a large variation at the individual level, all patient safety climate scores varied noticeably at the work site level. Since our model is simple, the estimated ICCs are less likely to be biased [29]. The result is consistent with results from a Scottish study that showed significant variation in safety climate between practice teams in primary care [6]. For all five patient safety climate dimensions in our study the two level models produced lower AIC values than the models ignoring the possibility of factor score variation across work sites. This indicates that the two level models fit better to the data. The results suggest that the Norwegian version of the SAQ A is able to identify variation in staff perceptions of patient safety climate across work sites in Norwegian General Practitioner practices and Out-of-hour clinics. Accordingly, some work sites may be more promising candidates for patient safety improvement interventions than others, for example, work sites where employees feel reluctant to speak up if they experience problems in patient care or perceive that their input is not wanted. The results reveal opportunity for leaders to improve behavior and results in their organisation by facilitating dialogue to strengthen trust, mutual values and relationships within groups of employees at work sites, and not only by influencing individuals[7, 30]. Although staff attitudes are strongly modified by work place culture, individual characteristics may also contribute[31]. Therefore, we explored individual characteristics related to patient safety climate scores in a previous paper [22]. Older health professionals scored higher than younger professionals, and female GPs scored significantly lower than male GPs. Knowing that patient safety climate perceptions are perceived significantly more positively by staff in leadership positions than their subordinates it is not unreasonable to think that age may have a similar effect [6, 22, 32]. Age may be associated with more experience, qualifications and confidence, which may influence staff to respond more positively to items like: “It is easy for personnel in this clinic to ask questions when there is something that they do not understand”, and “I know the proper channels to direct questions regarding patient safety in this clinic”. A potential strategy to encourage younger staff to be open about hazards and adverse events could be senior staff offering to arrange regular dialogue meetings and expose their own experiences of vulnerability in relation to hazards and adverse events. It is possible that female GPs scored significantly lower than male GPs because female GPs may identify more risks than male GPs [22]. The interpretation is supported by a German study that found that female medical doctors cared better for type 2 diabetes patients than male medical doctors [33]. A study from the US found that elderly hospitalised patients had lower mortality and readmission rates when treated by female internists compared to those treated by male medical doctors [34]. In trying to explain the gender difference, listening and communication skills, as well as spending more time with patients, were suggested as possible factors[35]. Such characteristics may also be relevant to explore in dialogue meetings where patient safety climate scores are discussed for improvement purpose.

For Job satisfaction, the organisational level variance had an ICC of 7.1%, which was lower than for the other factors (Table 1). This could mean that Job satisfaction in primary care in Norway is generally perceived as good and varies little by local work site conditions, how leaders and employees interact and how they are organised. We have previously published that support staff reported significantly higher job satisfaction in OOH clinics compared to GP practices and that job satisfaction achieved highest mean score of the five factors for both medical doctors and support staff in both GP offices and OOH clinics [22]. Our finding are consistent with a previous study, which revealed high job satisfaction amongst Norwegian GPs [36]. Norwegian GPs and psychiatrists reported significantly higher job satisfaction compared to other medical doctors, whilst Norwegian medical doctors also reported significantly higher job satisfaction than US medical doctors[37, 38]. In both countries job satisfaction was related to the medical doctors perceptions of quality of care [38]. The difference between the countries was partly ascribed to the health care systems, which demanded time and costs from the US medical doctors to arrange care in cases of limited healthcare coverage [39].

A limitation of our study is that it was conducted in only 23 clinics and practices. Based on a simulation study that recommended more than 30 on a general basis, our ICCs may be moderately overestimated [29]. The number of groups in our study is however far larger than 10, which, according to Snijders and Bosker, makes multilevel modelling attractive [26]. Although the numbers of clinics and practices in our study were fairly low, it was performed in a representative sample of OOH clinics in Norway in addition to a majority of GP practices of one county. The choice to invite all the GP practices of one county as based on the expectation that a majority of GP practices in the same county would be more representative with more variation in safety climates, compared to a minority of GP practices recruited across the whole country.

A second limitation is that the overall response rate of 52% was rather low. It was however almost twice as high amongst support staff (72%) than among medical doctors (39%). The rather low response rate of OOH medical doctors does not necessarily reduce the validity of the patient safety assessments in these clinics, as the support staff who commonly work more permanently in OOH clinics had a high response rate of 71%. Support staff who attend the work site daily generate a substantial experience of its climate and were well represented amongst the respondents in the study. The response rate was higher amongst medical doctors in GP practices (55%) than medical doctors in OOH clinics (33%). An explanation may be that medical doctors in GP practices may possibly be more interested in contributing to the evaluation of their work environment than OOH medical doctors. Most OOH medical doctors are GPs who work only parttime in the OOH setting and for relatively few hours in OOH clinics, as their main job is in the GP practice.

Based on our results, we suggest that patient safety improvement work in GP practices and OOH clinics should not only address all work sites in the same way, but focus on site specific challenges at places with lower scores on specific patient safety climate factors. That can be accomplished by creating opportunities for dialogue regarding quality of care at the specific sites, like for example quality team meetings. Such dialogue opportunities are associated with better safety climate scores [7, 30].

## Conclusions

Our results show that there was quite a bit of response clustering within the GP and OOH units. This implies that the Norwegian SAQ A is able to identify GP practices and OOH clinics with high and low patient safety climate scores. Patient safety climate scores produced by the Norwegian version of the SAQ A may, thus, guide improvement and learning efforts to work sites according to the level of their scores. Some units scored better, others scored worse. By discussing patient safety climate survey results, staff in lowscoring units and their leaders may identify opportunities for improvement and develop their understanding of how to reduce risks of adverse events and to improve patient safety.

## Supporting information

S1 Table The five patient safety climate factors and corresponding items confirmed in the validated Norwegian translation of the Safety Attitudes Questionnaire–Ambulatory Version (SAQ-AV). Respondents rate their agreement using a 5-point Likert scale: 1 = disagree strongly, 2 = disagree slightly, 3 = neutral, 4 = agree slightly, 5 = agree strongly.
(DOCX)

S1 File Data file SAQ GP clinics PLOS ONE. (SAV)

S2 File Data file SAQ OOH clinics PLOS ONE. (SAV)
