# Supplementary material for: Variation in staff perceptions of patient safety climate across work sites in Norwegian general practitioner practices and out-of-hour clinics
Source: PLoS One. 2019 Apr 10;14(4):e0214914. doi: 10.1371/journal.pone.0214914 (PMC6457548; doi:10.1371/journal.pone.0214914)
Supplement: S1 Table — Respondents rate their agreement using a 5-point Likert scale: 1 = disagree strongly, 2 = disagree slightly, 3 = neutral, 4 = agree slightly, 5 = agree strongly. (DOCX) [file pone.0214914.s001.docx]

**S1 Table.** **The five patient safety climate factors and corresponding items confirmed in the validated Norwegian translation of the Safety Attitudes Questionnaire – Ambulatory Version (SAQ-AV).** Respondents rate their agreement using a 5-point Likert scale: 1 = disagree strongly, 2 = disagree slightly, 3 = neutral, 4 = agree slightly, 5 = agree strongly.

| **Teamwork climate** | Nurse input is well received in this office. |
| --- | --- |
|  | In this office, it is difficult to speak up if I perceive a problem with patient care. |
|  | Disagreements in this office are resolved appropriately (i.e., not *who* is right but *what* is best for the patient). |
|  | I have the support I need from other personnel to care for patients. |
|  | It is easy for personnel in this office to ask questions when there is something that they do not understand. |
|  | The physicians and nurses here work together as a well-coordinated team. |
|  | During emergencies, I can predict what other personnel are going to do next. |
|  | I am frequently unable to express disagreement with staff physicians/ intensivists in this office. |
|  | Attending physicians/ primary care providers in this office are doing a good job. |
| **Safety climate** | I would feel safe being treated here as a patient. |
|  | Medical errors are handled appropriately in this office. |
|  | I receive appropriate feedback about my performance. |
|  | In this office, it is difficult to discuss errors. |
|  | I am encouraged by my colleagues to report any patient safety concerns I may have. |
|  | The culture in this office makes it easy to learn from the errors of others. |
|  | I know the proper channels to direct questions regarding patient safety in this office. |
| **Working conditions** | This office does a good job of training new personnel. |
|  | All the necessary information for diagnostic and therapeutic decisions is routinely available to me. |
|  | This office deals constructively with problem personnel. |
|  | Trainees in my discipline are adequately supervised. |
| **Job satisfaction** | I like my job. |
|  | Working in this office is like being part of a large family. |
|  | This office is a good place to work. |
|  | I am proud to work at this office. |
|  | Morale in this office is high. |
| **Perceptions of management** | The management of this office supports my daily efforts. |
|  | Office management does not knowingly compromise the safety of patients. |
|  | The levels of staffing in this office are sufficient to handle the number of patients. |
|  | I am provided with adequate, timely information about events in the office that might affect my work. |
